# Risk factors for inducing violence in patients with delirium

**DOI:** 10.1002/brb3.2276

**Published:** 2021-08-02

**Authors:** Masako Tachibana, Toshiya Inada, Masaru Ichida, Norio Ozaki

**Affiliations:** ^1^ Department of Psychiatry Nagoya Ekisaikai Hospital Nagoya‐shi Aichi Japan; ^2^ Department of Psychiatry Nagoya University Graduate School of Medicine Nagoya‐shi Aichi Japan; ^3^ Department of Psychobiology Nagoya University Graduate School of Medicine Nagoya‐shi Aichi Japan

**Keywords:** delirium, ICU, male, risk factors, smoking, violence

## Abstract

**Background:**

Violence in patients with delirium may occur suddenly and unpredictably in a fluctuating state of consciousness. Although various factors are involved, appropriate assessment and early response to factors related to violence in delirium are expected to prevent dangerous and distressing acts of violence against patients, their families and medical staff, and minimize the use of physical restraint and excessive drug sedation.

**Methods:**

Subjects were 601 delirium cases referred to the department of psychiatry over the course of 5 years at a general hospital. The demographic, clinical, and pharmacological variables of patients with violence (*n* = 189) were compared with those of patients without violence (*n* = 412). Logistic regression analysis was applied to determine whether any specific individual factors were associated with violence.

**Results:**

Current smoker status (*p *< .0005), older age (*p* < .0005), male gender (*p* = .004), and use of intensive care units (*p* = .043) were identified as factors associated with violence in patients with delirium.

**Conclusions:**

Screening tools for violence in patients with delirium and adequate management may assist in better outcomes for patients and medical staff. Further research should evaluate the usefulness of nicotine replacement treatment for the prevention of violence during nicotine withdrawal, including whether it is safe for elderly inpatients with a high incidence of delirium in clinical practice.

## 1 INTRODUCTION

In delirium, the patient is unable to recognize that he or she is in a hospital and receiving treatment, and misperceptions of the situation (such as “Strangers are trying to inflict harm upon me”), combined with psychomotor agitation, may lead to violent acts such as hitting, kicking, and biting the nurse in intense resistance (resisting behaviors; Cipriani et al., 2011; Wharton et al., 2018). For patients in a state of confusion and fear, violence too may be an act of self‐preservation (defensive behaviors; Cipriani et al., 2011; Wharton et al., 2018). Inpatients with delirium have been reported as 11 times more likely to be associated with an increased risk of incidents of aggression when compared to general inpatients (Williamson et al., 2014). Delirium is a rapidly fluctuating condition that occurs over a short period of time (American Psychiatric Association, 2013); therefore, violence can appear suddenly and unpredictably. As a result, physical restraint or drug sedation may have to be considered. However, these can often lead to a vicious cycle. Physical restraint can cause further anxiety and distress, distrust, and anger toward medical staff, as well as increase the persistence of delirium and risk of injury (Inouye et al., 2014; Marcantonio, 2017). Antipsychotics are often used against agitation and violence in patients with delirium in clinical practice, but most studies do not show any signs of its effectiveness in decreasing the severity or duration of delirium, and they may even contribute to heightened adverse effects and poor long‐term outcomes (Oh et al., 2017). The initial assessment and early identification of patients at risk of violence should begin as soon as the patients develop delirium. Appropriate assessment of factors related to violence, and its management, are expected to prevent dangerous and distressing acts of violence against patients, their families, and medical staff, as well as minimize physical restraint and excessive drug sedation. However, little is known about the risk factors for violence in patients with delirium.

In the present study, various factors, including demographic, clinical, and pharmacological aspects, were examined for association with violence in patients with delirium to identify risk factors. Our findings will potentially lead to appropriate treatment and care of delirium in clinical practice.

## 2 MATERIAL AND METHODS

### 2.1 Ethical considerations

This study was initiated after the approval of the ethics committee of the Nagoya Ekisaikai Hospital, and was carried out in accordance with the Helsinki Declaration of 1964 and its later versions.

### 2.2 Procedure

The survey involved inpatients of Nagoya Ekisaikai Hospital, a general hospital with 602 beds, containing 54 beds for the emergency center, and no psychiatric beds. During the past 5 years, from May 2015 to August 2020, 601 delirium patients referred to the department of psychiatry were the subjects of the current analysis, after excluding some patients due to lack of data. Delirium was diagnosed based on the 5th edition of the *Diagnostic and Statistical Manual of Mental Disorders* (*DSM‐5*) (American Psychiatric Association, 2013), by two full‐time psychiatrists. Violence refers to actions that inflict physical harm in violation of social norms. The following clinical variables were extracted from the medical records: sex, age, body mass index (BMI), medical complications before admission (visual and hearing impairments, hypertension, diabetes mellitus, cardiovascular disease, chronic obstructive pulmonary disease (COPD), cerebrovascular disorders, dementia, depressive disorders, and anxiety disorders), smoking status, alcohol consumption before admission, whether patients have stopped taking benzodiazepines (BZDs) after admission (BZDs withdrawal), presence or absence of surgery (cardiosurgery and non‐cardiosurgery), general anesthesia, whether patients were in intensive care units when delirium developed, and finally, pharmacological treatment after admission. The variable of pharmacological treatment included: corticosteroids, opioid analgesics, anticholinergics, histamine 1 (H_1_) receptor blockers, histamine 2 (H_2_) receptor blockers, selective serotonin reuptake inhibitor and serotonin noradrenaline reuptake inhibitor (SSRI and SNRI), calcium (Ca) channel blockers, β‐blockers, Angiotensin‐converting enzyme (ACE) inhibitors, Angiotensin II receptor blockers (ARBs), antiarrhythmic drugs, digitalis, dopamine receptor agonists, non‐steroidal anti‐inflammatory drugs, antibiotics, antifungals, antiprotozoal drugs, anticonvulsants, cholinesterase inhibitors, and hypnotics (BZDs, suvorexant, and ramelteon). These include (1) medications that the patients had been receiving before hospitalization, and continued receiving after hospitalization, and (2) those newly prescribed that the patients had been receiving after hospitalization. Among the above variables, age and BMI were analyzed as continuous variables, alcohol consumption (0, none; 1, habitual; 2, heavy; 3, dependence) and smoking status (0, none; 1, habitual; 2, heavy; patients who quit smoking prior to admission for scheduled surgery were classified as “none”) were analyzed as categorical variables, and the others were dummy variables. The above variables used to examine their association with violence in patients with delirium were chosen on the basis of previous reports and clinical relevance.

### 2.3 Statistics

All statistical analyses (Chi‐square test and logistic regression analysis) were performed using the IBM SPSS statistical software ver. 27.0 (IBM Corp., Armonk, NY, USA). Significant difference was set at *p *< .05.

## 3 RESULTS

### 3.1 Demographic and clinical characteristics of the subjects

Of the 601 patients who developed delirium, the average age was 75.8 years (standard deviation, 12.2; age range, 24.3−100.9 years), 56% of the patients were male, and the mean body mass index (BMI) was 21.6 kg/m^2^. There were 189 patients with violence, and 412 patients without violence; the incidence of violence was 31.4%. Table 1 shows the patients’ demographic and clinical characteristics, as well as Pearson's correlation coefficients between these variables and violence observed. Table 2 shows the prescribed medications patients were receiving during the development of delirium, as well as Pearson's correlation coefficients between these variables and violence observed.

**TABLE 1:** Demographic and clinical characteristics of the patients with delirium

Demographic and clinical variables	Patients without violence	Patients with violence	Pearson's correlation	*p*‐Value
Number of patients (%)	412 (69%)	189 (31%)		
Male ratio [%]	52%	67%	.126	<.0005
Mean age [years] (SD)	74.8 (12.7)	78.2 (10.7)	.137	.001
Average BMI [kg/m^2^] (SD)	21.4 (4.2)	21.8 (4.4)	.039	.17
Rate of patients with a current smoking habit			.153	<.0005
Habitual (20 cigarettes or less)	15%	24%		
Heavy (more than 20)	3%	6%		
Rate of patients drinking alcohol			−.026	.259
Habitual	20%	28%		
Heavy	7%	6%		
Dependence	9%	4%		
Rate of patients with the following complications				
Visual impairment	46%	41%	−.043	.148
Hearing impairment	23%	28%	.042	.153
Hypertension	53%	61%	.074	.035
Diabetes	25%	25%	−.002	.481
Cardiovascular disease	11%	11%	−.007	.428
Chronic obstructive pulmonary disease	6%	9%	.040	.162
Cerebrovascular disorders	18%	20%	.024	.276
Dementia	46%	49%	.027	.253
Depressive disorders	6%	3%	−.076	.031
Anxiety disorders	2%	1%	−.047	.125
Rate of patients who have stopped taking BZDs after admission (BZD withdrawal)	22%	21%	−.009	.412
Rate of patients receiving cardiac surgery before delirium developed during hospitalization	9%	7%	−.021	.301
Rate of patients receiving non‐cardiac surgery before delirium developed during hospitalization	21%	23%	.032	.218
Rate of patients receiving general anesthesia before delirium developed during hospitalization	25%	25%	.006	.443
Rate of patients who were in intensive care units when delirium developed	49%	59%	.091	.013

Abbreviations: BZD, benzodiazepine; SD, standard deviation; min‐max, minimum‐maximum.

**TABLE 2:** Prescribed medications in patients with delirium

Type of medication	Patients without violence	Patients with violence	Pearson's correlation	*p‐*Value
Corticosteroids	13%	10%	−.050	.111
Opioid analgesics	22%	24%	.024	.280
Anticholinergics^a^	25%	23%	−.020	.314
Histamine 1 (H_1_) receptor blockers	8%	7%	−.015	.360
Histamine 2 (H_2_) receptor blockers	13%	11%	−.017	.340
SSRIs / SNRIs	2%	2%	−.002	.484
Calcium (Ca) channel blockers	35%	41%	.063	.060
b‐blockers	20%	22%	.026	.265
Angiotensin‐converting enzyme inhibitors	6%	6%	−.004	.462
Angiotensin II receptor blockers	14%	15%	.011	.392
Antiarrhythmic drugs^b^	4%	5%	−.021	.302
Digitalis	1%	2%	.016	.352
Dopamine receptor agonists	2%	2%	.006	.439
Nonsteroidal anti‐inflammatory drugs	28%	31%	.031	.226
Antibiotics	48%	50%	.024	.282
Antifungals	1%	1%	.004	.457
Antiprotozoal drug	1%	0%	−.048	.121
Anticonvulsants	4%	5%	.010	.407
Cholinesterase inhibitors	5%	7%	.047	.126
Benzodiazepines	59%	55%	−.040	.162
Suvorexant	16%	23%	.089	.014
Ramelteon	11%	10%	−.024	.283

^a^ Compounds that have anticholinergic activity, such as H_1_ blockers, H_2_ blockers, dopamine receptor agonists, SSRI and SNRI, are not included in the “anticholinergics.”

^b^ Compounds that have antiarrhythmic activity, such as b‐blockers and calcium channel blockers, are not included in the “antiarrhythmic drugs.”

Abbreviations: SSRI, selective serotonin reuptake inhibitor; SNRI, serotonin noradrenaline reuptake inhibitor.

### 3.2 The features of violence

All of the violence was targeted at the medical staff (mostly nurses), and occurred in situations involving personal care, such as medical procedures and bed‐bath, and no violence against family members or other inpatients was documented in the patients in this study. The violent behaviors included hitting, kicking, biting, punching, pinching, shoving, hard grabbing, throwing objects, spitting, and scratching. Two types of symptoms that preceded violence were identified: impulsivity (impulsivity‐dominant type) and ideations or delusions of persecution by medical staff (delusion‐dominant type). Males were significantly more associated with the impulsivity‐dominant type, and females with the delusion‐dominant type (χ^2 ^= 5.889, df = 2, *p* = .015).

### 3.3 Risk factors for inducing violence in patients with delirium

Logistic regression analysis was performed to determine which factors were associated with violence. Of the 22 demographic and clinical variables shown in Table 1, and the 22 kinds of medication variables shown in Table 2, a total of 17 variables, whose *p* values were less than .2, were selected as independent variables. The results showed that current smoker status (*p* < .0005), older age (*p* < .0005), male gender (*p* = .004), and use of intensive care units (*p* = .043) were identified as factors associated with violence in patients with delirium (Table 3); no other variables were. It is to be noted that none of the patients who quit smoking prior to admission for scheduled surgery (*n* = 6) showed violence.

**TABLE 3:** Variables significantly associated with violence in the patients with delirium

Variables	Odds ratio	*p*‐Value	95% CI
Current smoker status	2.64	.000	1.75–3.40
Older age	1.06	.000	1.04–1.08
Male gender	1.88	.004	1.23–2.88
Use of intensive care units	1.51	.043	1.01–2.26

Abbreviation: CI, confidence interval.

## 4 DISCUSSION

In the present results, the incidence of violence in patients with delirium was 31.4%. Current smoker status, older age, male gender, and use of intensive care units were identified as factors associated with violence in patients with delirium. In previous reports, the incidence of aggression was 10.8% (Williamson et al., 2014), while the incidence of aggressive behavior was 25.8% (Wharton et al., 2018) in patients with delirium. The discrepancy may reflect differences in the subjects of analysis. In general, among cases with delirium, those who show extremely severe motor agitation are usually referred from other departments to the psychiatric department in general hospitals. A number of milder cases of delirium were not referred to the psychiatric department, and these cases were not included in this study.

Current smoking was shown to increase the incidence of violence in patients with delirium. In a previous study, daily smokers in a community‐based sample have been reported to be 2.1 times more likely than non‐smokers to display violence (Lewis et al., 2016). The habit of smoking has been recognized as a risk factor for agitation due to the risk of withdrawal syndrome, direct neurotoxic effect, or deterioration of pulmonary function (Almeida et al., 2016; Kang et al., 2020; Park et al., 2016). The present study showed that current smoker status is significantly associated with violence, even after removing the effects of biological confounders such as gender and COPD. In clinical practice, it seems that not as much attention is given to nicotine withdrawal compared to alcohol withdrawal. Although the smoking rate has been decreasing in Japan, among adult males it is at 27.1% and 23.1% among those over 60 years of age; still high compared to other countries (Ministry of Health, Labor and Welfare). Further research is needed, including the extent of smoking, such as its duration and total amount, to quantify baseline smoking status and risk of nicotine withdrawal. In previous studies, the evidence for the use of nicotine replacement treatment (NRT) in agitation and delirium management has been inconclusive due to a paucity of high‐quality data (Kowalski et al., 2016). Recently, with a randomized controlled pilot study of NRT in the intensive care unit, the safety and efficacy of NRT for the prevention of delirium were reported (de Jong et al., 2018). The efficacy of NRT in the prevention of violence for high‐risk patients of nicotine withdrawal, such as heavy smokers, including the safety of its application in elderly inpatients with delirium, should be further evaluated.

Older age has been reported to be associated with aggression and violence in inpatients (Ideker et al., 2011; Williamson et al., 2014). In the present study, older age and use of ICU were identified as significant factors of violence in patients with delirium. Older age may be related to the degree of severity of delirium and/or dementia, and it has been suggested that patients with dementia are particularly sensitive to stress‐provoking factors within their physical environment, and may react to these factors with challenging behavior such as aggression (Cipriani et al., 2011). Especially in unfamiliar and irritating general hospital settings like the ICU, where the incidence of severe delirium is also high, violence may result from severe confusion and difficulties in understanding the situation. In addition, situations involving personal care have been associated with higher levels of aggression, suggesting that the patient misinterprets such care as a personal violation and intrusion of personal space (Cipriani et al., 2011). In this study as well, all violence was directed at the medical staff (mostly nurses) and occurred in situations involving personal care, such as medical procedures and bed‐bath. Explanation of the situation and informed consent for diagnosis and treatment, need to be done repetitively and clearly to the patient with delirium.

Male gender was found to contribute to violence in patients with delirium. A number of previous studies have reported that men are more likely to behave aggressively than women, and that men's aggression is expressed in a more physical way (Almeida et al., 2016; Cipriani et al., 2011; Ideker et al., 2011; Williamson et al., 2014), and the same was observed in delirium in this study.

As per a previous study, a history of mental health conditions such as depression or anxiety may lead to an increased probability of aggressive behaviors (Wharton et al., 2018), but this could also be caused by the influence of drugs such as SSRIs, SNRIs, and BZDs which are used for depression and anxiety. In the present study, history of depressive and anxiety disorders, and use of SSRIs/SNRIs/BZDs were not associated with violence in delirium.

Several limitations could be considered in this study. First, this was a retrospective study based on clinical practice at a single hospital. Further studies on the subjects of other general hospitals will help bolster the present results. Second, the present subjects were receiving various kinds of medications, some of which were not included in this analysis. More detailed research such as drug interaction and dose‐finding effects will be needed, including the various medications excluded in the present study. Third, the data on physical variables such as pain, psychological variables such as anxiety, uncomfortable and painful medical procedures such as urethral drainage tubes, and behavioral disturbance history, which could be factors for violence, were not included in this study.

In conclusion, regardless of the limitations mentioned above, this is one of the largest studies investigating the risk factors for violence in patients with delirium in a general hospital. While a complex range of biological factors (older age and male gender), environmental factors (use of ICU), and withdrawal of exogenous substances (current smoking status) played significant roles, screening tools for violence in patients with delirium, and adequate management such as consideration for environmental factors and treatment of nicotine withdrawal, could reduce the incidence of violence, physical restraint, and excessive drug sedation in patients with delirium and assist in better outcomes for both patients and the medical staff. Further research should evaluate the usefulness of NRT in the prevention of violence during nicotine withdrawal, including whether it is safe for elderly inpatients with a high incidence of delirium in clinical practice.

## CONFLICT OF INTEREST

Dr. N. Ozaki has received research support or speakers’ honoraria from, or has served as a consultant to, Sumitomo Dainippon, Otsuka, DAIICHI SANKYO, Tsumura, Nihon Medi‐Physics, Pfizer, KAITEKI, Eli Lilly, Mochida, Eisai, Takeda, Novartis, Astellas, MSD, Meiji Seika Pharma, Janssen, Mitsubishi Tanabe, Kyowa, Taisho Pharma, EA Pharma, UCB, and Shionogi, outside the submitted work. Other authors declare no conflict of interest for the publication of this study.

## AUTHOR CONTRIBUTIONS

All authors conceived and designed the study. Masako Tachibana and Masaru Ichida performed the data collection. Masako Tachibana and Toshiya Inada conducted the statistical analysis and wrote the first draft of the manuscript. All authors revised and approved the final version of the manuscript.

## Data Availability

The data that support the findings of this study are available from the corresponding author upon reasonable request.
